# Characterization of NLRP3 Inflammasome‐Associated Hub Genes in the Progression of Diabetic Nephropathy

**DOI:** 10.1002/iid3.70424

**Published:** 2026-04-13

**Authors:** Sheng Zhao, Yuejiao Li, Wenchuan Li, Lan Dong, Rong Lian, Qingqing Luo, Xingji Lian, Jianbo Li, Feng He

**Affiliations:** ^1^ Department of Nephrology, Guangzhou First People's Hospital, The Second Affiliated Hospital, School of Medicine South China University of Technology Guangzhou China; ^2^ Clinical Research Center, The Second Affiliated Hospital, School of Medicine South China University of Technology Guangzhou China; ^3^ Department of Gastroenterology and Hepatology, Guangzhou First People's Hospital, School of Medicine South China University of Technology Guangzhou China; ^4^ Department of Geriatrics, Guangzhou First People's Hospital The Second Affiliated Hospital of South China University of Technology Guangzhou China; ^5^ Department of Nephrology, Key Laboratory of Nephrology, The First Affiliated Hospital, Ministry of Health Sun Yat‐Sen University Guangzhou China

**Keywords:** diabetic nephropathy, IL‐1β, inflammation, NLRP3, WGCNA

## Abstract

**Background:**

Diabetic nephropathy (DN) is widely recognized as the primary cause of end‐stage renal disease. However, the underlying mechanisms and pathogenesis of DN remain incompletely understood. Exploring novel biomarkers aiding in tracking the progression of DN has important clinical implications.

**Methods:**

Human renal transcriptomic datasets (GSE142025 and GSE96804) from the Gene Expression Omnibus (GEO) were analyzed. Differentially expressed genes (DEGs) were identified using a staged analysis, which involved sequential comparisons between normal controls and early‐stage DN, as well as between early DN (eDN) and advanced DN (aDN). Enrichment analysis of DEGs was performed using R software. WGCNA was utilized to construct gene co‐expression networks and to identify the key genes. Then, Venn diagrams were generated using DEGs and key genes from both datasets to determine the final hub genes. ROC curves were then used to assess the diagnostic accuracy of hub genes for DN disease progression. Finally, we cross‐validated the hub genes using the clinical kidney specimens.

**Results:**

After analyzing the datasets, we identified 22 and 43 hub genes using the WGCNA and DEGs at the eDN and aDN stages. Further investigation of the literature on the hub genes led to the discovery of their association with the activity of NLRP3 inflammasome, including *ZFP36*, *CLEC2D*, and *HCK*, which were identified as NLRP3 inflammasome‐associated hub genes (NIAHGs). We found that the AUC of all the NIAHGs can indicate their potential diagnostic value. Then, we validated the expression levels of NIAHGs in human renal tissues, which were consistent with those in both data sets. Importantly, immunoblot analysis indicated that NIAHGs expression was associated with NLRP3 inflammasome activation. Furthermore, NLRP3 inflammasome was significantly activated, leading to the release of large amounts of IL‐1β and IL‐18 in eDN, further initiating the inflammatory response in the diabetic kidney.

**Conclusions:**

Our findings identify critical hub genes associated with DN progression and NLRP3 inflammasome activity, providing a theoretical basis and candidate targets for subsequent research.

## Graphical abstract

Expression of molecules related to the activation of NLRP3 inflammasomes and NIAHGs. (A) Immunoblot analysis of NLRP3, pro‐caspase‐1, caspase‐1 p20, and NIAHGs proteins in renal tissues from NC, eDN, and aDN patients. (B–E) Relative IL‐1β and IL‐18 levels across groups determined by RT‐qPCR (B, C) and ELISA (D, E). *n* = 6, three technical replicates. Data are mean ± SD. *p < 0.05; **p < 0.01; ***p < 0.001; and ****p < 0.0001. Significance determined by one‐way ANOVA followed by Tukey's post hoc test.

## 1 Introduction

Diabetic nephropathy (DN) is the most prevalent microvascular complication of diabetes and remains the primary cause of end‐stage renal disease (ESRD) [1, 2]. The escalating morbidity and mortality rates associated with DN impose a significant and ongoing medical burden on individuals, families, and society [3, 4]. In the early stages of DN, some loss of kidney function is reversible with early intervention and effective diabetes management. However, as the disease progresses, the structure and function of the kidneys may be permanently damaged, making it difficult to reverse through intervention [1]. Currently, routine diagnosis and prognostic assessment of DN is based primarily on the degree of albuminuria and decline in renal function. However, there is considerable inter‐individual variability in the occurrence of albuminuria [5, 6]. Although renal biopsy can be used for the pathological diagnosis of DN, its significant associated risks limit its clinical application. To date, epidemiologic studies and existing diagnostic methods have been unable to definitively indicate the detection and progression of early DN (eDN). Hence, identifying novel biomarkers in the progression of DN patients can facilitate improved diagnostic accuracy and timely therapeutic intervention for the disease, thereby delaying the approach of ESRD.

DN is perceived as a complex disease caused by various factors, including genetics, hemodynamic changes, metabolic disorders, oxidative stress, and inflammatory responses [7, 8, 9, 10]. Compelling evidence supports the pivotal role of systemic and local inflammation in the progression of DN, predominantly characterized by immune cell infiltration and increased expression of inflammatory cytokines and chemokines [11]. In a hyperglycemic milieu, the NF‐κB signaling pathway is activated [12]. Concurrently, this pathway promotes activation of the NLRP3 inflammasome, which initiates the synthesis and release of IL‐1β and IL‐18 [13]. A growing body of evidence suggests a dynamic involvement of the NLRP3 inflammasome in the pathogenesis of DN [14]. In particular, deficiency of NLRP3 or caspase‐1 has been shown to attenuate inflammatory injury and fibrosis within DN [15].

Recently, there has been significant progress in microarray technology and bioinformatics analysis. RNA expression profiles have been mapped using omics techniques, providing insights into changes in thousands of genes throughout the disease process [16, 17]. Here, our investigation used a spectrum of bioinformatics methods to explore hub genes that are critical to the progression of DN. We then verified the expression levels of the hub gene NLRP3 and its activation markers in human tissues. Our results suggest a potential correlation between hub genes and NLRP3 activation in DN progression. These findings indicate a possible mechanism by which hub genes contribute to the progression of DN.

## 2 Materials and Methods

### 2.1 Data Collection and Preparation

Two public gene expression datasets, GSE142025 and GSE96804, were downloaded from the Gene Expression Omnibus (GEO) database for analysis (http://www.ncbi.nlm.nih.gov/geo). The GSE142025 dataset includes 28 biopsy‐proven DN patients, comprising 6 with early‐stage DN and 22 with advanced‐stage DN. Control samples (*n* = 9) were obtained from unaffected portions of tumor resection specimens [18]. The GSE96804 dataset includes 41 patients diagnosed with DN via renal biopsy, comprising 20 with early‐stage DN and 21 with advanced‐stage DN. Control samples (*n* = 20) were obtained from biopsy specimens of unaffected portions of tumor nephrectomy tissues [19]. eDN was defined by urinary albumin‐to‐creatinine ratio (UACR) between 30 and 300 mg/g and estimated glomerular filtration rate (eGFR) > 90 mL/min/1.73 m², while advanced DN (aDN) was defined by UACR > 300 mg/g and eGFR < 90 mL/min/1.73 m². All patients with DN have type 2 diabetes. Detailed clinical characteristics of the patients included in these datasets are provided in the Supporting Information (Table S1).

### 2.2 Identification and Analysis of Differentially Expressed Genes (DEGs)

In this study, we investigated differential mRNA expression in two datasets, GSE142025 and GSE96804, using the limma package of the R software. We employed the Bayesian multiple‐testing correction to screen for DEGs in GSE142025, with |log2FC| > 0.5 and *p* < 0.05. Similarly, we screened for DEGs in GSE96804 with |log2FC | > 0.3 and *p* < 0.05. We calculated DEGs between the eDN and normal control (NC) groups as well as between the aDN and eDN groups separately. Volcano maps were generated using the ggplot2 package. The complete list of DEGs has been deposited in Figshare (DOI:10.6084/m9.figshare.30489911; https://figshare.com/).

### 2.3 GO Enrichment Analysis and KEGG Pathway Analysis of DEGs

To investigate the functional roles and signaling pathways associated with DEGs, we performed GO functional enrichment and KEGG signaling pathway enrichment analyses on the DEGs, with a significance threshold of *p* < 0.05, to determine statistically significant results.

### 2.4 Building Gene Co‐Expression Networks and Discovering Gene Modules Associated With DN

We employed the WGCNA package in R to construct a co‐expression network for the datasets GSE142025 and GSE96804. Before analysis, we used the “impute” package in R to perform quality assessment, which involved identifying and removing outliers using sample clustering maps. The selection of a soft threshold power β value (ranging from 1 to 20) was then determined using the pickSoftThreshold function of the WGCNA package to ensure that the network adhered to a scale‐free distribution (standard *R* ^2^ > 0.9) at the chosen threshold (Figures S2 and S3). Subsequently, we conducted a Pearson correlation analysis using the soft threshold β to determine the correlation matrix between all gene pairs. We converted the correlation matrix to an adjacency matrix and then utilized it to calculate the topological overlap matrix (TOM) and its corresponding heterozygosity matrix (1‐TOM). Subsequently, we constructed a hierarchical clustering dendrogram to classify genes into distinct modules based on their expression similarity. We then used Module Eigengenes (ME) to summarize the expression profile of each module and calculated and visualized correlations between ME and clinical features. We focused on modules displaying high correlation coefficients with clinical features and selected genes as key genes from these modules for further analysis.

### 2.5 Identification of Hub Genes

We calculated the relationship between genes and ME, as well as gene significance (GS) in the focal module. The key genes within the module were selected and visualized based on the criteria of a correlation coefficient of MM > 0.8 and GS > 0.2. The DEGs and key genes from both datasets were used to generate a Venn diagram, where the intersection represented the final hub genes.

### 2.6 Human Kidney Biopsy Collection

A total of 18 human kidney tissue samples were obtained at Guangzhou First People's Hospital. Among these, 12 were renal biopsy specimens from patients with DN, and 6 were normal control kidney tissues derived from histologically normal regions of nephrectomy specimens resected for localized renal tumors. For DN patients, inclusion criteria were: (1) type 2 diabetes with biopsy‐confirmed DN; (2) age 18–80 years; (3) complete clinical and pathological data. Exclusion criteria were: (1) other glomerular diseases or systemic autoimmune disorders; (2) malignant tumors; (3) nephrotoxic drug use within the last 3 months; (4) pregnancy or lactation. Eligible DN patients were stratified into eDN (UACR 30–300 mg/g, eGFR > 90 mL/min/1.73 m², *n* = 6) and aDN (UACR > 300 mg/g, eGFR < 90 mL/min/1.73 m², *n* = 6) based on UACR and eGFR [18]. The clinical and pathological characteristics of the patients are detailed in Table S6. Renal biopsy was performed under ultrasound guidance after obtaining written informed consent. Specimens were collected from the lower pole of the kidney parenchyma, with the puncture site selected at the midpoint between the lower margin of the renal collecting system and the lower renal border. The needle was inserted perpendicularly through the renal capsule to ensure adequate tissue acquisition. Upon collection, each tissue sample was divided into portions and either fixed in paraformaldehyde for histological sectioning or snap‐frozen in liquid nitrogen for RNA and protein extraction. This study was approved by the Ethics Committee of Guangzhou First People's Hospital (Approval No. K‐2022‐014‐01). Written informed consent was obtained from all participants prior to inclusion.

### 2.7 Histological Evaluations

Formalin‐fixed paraffin‐embedded tissues were sectioned into 3 μm thickness. Histologic evaluation, HE, Masson trichrome, Periodic acid‐Schiff (PAS), and Periodic acid‐silver methenamine (PASM) staining were performed according to established and standardized protocols [21]. In PAS‐stained sections, 20 glomeruli were randomly selected from each kidney at 400× magnification for mesangial matrix analysis. For each glomerulus, the mesangial area was determined by quantifying the PAS‐positive and anuclear area in the mesangial region, and the proportion of the mesangial area to the total area of each glomerulus was statistically analyzed using Image‐Pro Plus 6.0 software (Image‐Pro Plus, Media Cybernetics Inc., USA) [22]. In the quantitative analysis of renal interstitial fibrosis using Masson's trichrome staining, six random fields of view were selected from each renal sample, and the proportion of the blue‐stained positive area was analyzed with the assistance of Image‐Pro Plus 6.0 software [23].

### 2.8 Validation of Hub Genes With ROC Curve Analysis

ROC curves were generated to evaluate the diagnostic accuracy of hub genes in disease progression using the expression data from the validation set GSE96804 and the “pROC” package. Hub genes with an area under the curve (AUC) greater than 0.6 were deemed clinically significant regarding their specificity and sensitivity for disease diagnosis. Moreover, the expression of hub genes in the GSE142025 dataset was extracted and visualized using scatter plots generated with the R “ggplot2” package to depict differential expression patterns of hub genes during disease progression.

### 2.9 RNA Expression Analysis and Sequences

Total RNA was extracted from kidney tissue using RNAiso plus reagent (TaKaRa) according to established and standardized protocols [24]. cDNA was acquired using NovoScript Plus All‐in‐one 1st Strand cDNA Synthesis SuperMix (Novoprotein) reagent and according to its manufacturer's instructions. cDNA was obtained using specific primers, 3 ng cDNA, and Ultra SYBR master mix (High ROX) (Cwbiotech) in QuantStudio5 (Thermo Fisher Scientific) for qRT‐PCR experiments. The relative expression of mRNA was calculated using the ∆∆Ct method. GAPDH was used as an internal reference gene. The following primer sequences were used: *ZFP36*: forward 5′‐GACTGAGCTATGTCGGACCTT‐3′ and reverse 5′‐GAGTTCCGTC TTGTATTTGGGG‐3′; *CLEC2D/LLT1*: forward 5′‐ATGCATGACAGTAACAATGTGG‐3′ and reverse 5′‐TAGTTGGGGCTTTGCTGTAA‐3′; *HCK*: forward 5′‐TCCTCCGAGATGGAAGCAAG‐3′ and reverse 5′‐ACAGTGCGACCACAATGGTAT‐3′; *IL‐1β*: forward 5′‐GTGGTGGTCGGAGATTCGTAG‐3′ and reverse 5′‐GAAATGATGGCTTATTACAGTGGC‐3′; *GAPDH*: forward 5′‐GGAGCGAGATCCCTCCAAAAT‐3′ and reverse 5′‐GGCTGTTGTCATACTTCTCATGG‐3′; *IL‐18*: forward 5′‐TCTTCATTGACCAAGGAAATCGG‐3′ and reverse 5′‐TCCGGGGTGCATTATCTCTAC‐3′.

### 2.10 Immunohistochemical Staining

The Ethics Committee of Guangzhou First People's Hospital approved this study. Biopsy specimens were embedded in paraffin, sectioned, and subjected to immunostaining with specific antibodies according to established and standardized protocols [25]. The following antibodies were used in this study: ZFP36 (12737‐1‐AP, Proteintech), CLEC2D (13188‐1‐AP, Proteintech), and HCK (11600‐1‐AP, Proteintech). The immunohistochemical images were captured using a 400× field of view. The images at other magnifications are presented in the Supporting Information (Figures S3 and S4). Quantitative analysis of immunohistochemically stained positive areas was performed using optical density measurements via Image‐Pro Plus 6.0 software. The same settings were used for each area, and the optical density was calibrated [26].

### 2.11 Western Blot Analysis

The protein concentration was determined by total protein extraction from kidney tissue using established methods [27]. Subsequently, the protein was separated through electrophoresis and electrotransferred onto PVDF membranes. The membranes were incubated with skimmed milk powder at room temperature for 1 h, followed by overnight incubation at 4°C with the specific primary antibodies. The membranes were then incubated with the corresponding secondary antibodies for 1 h at room temperature. Finally, chemiluminescence staining was used to detect protein expression levels, and protein blots were visualized using an image scanner. Following are the antibodies used in this study: ZFP36 (12737‐1‐AP, Proteintech), CLEC2D (13188‐1‐AP, Proteintech), HCK (11600‐1‐AP, Proteintech), NLRP3 (WL02635, Wanleibio), caspase‐1 (22915‐1‐AP, Proteintech), Vinculin (66305‐1‐Ig, Proteintech), β‐actin (AC026, ABclonal). The original image can be found in the Supporting Information (Figure S5).

### 2.12 Enzyme‐Linked Immunosorbent Assay (ELISA)

Evaluation of IL‐1β and IL‐18 in renal homogenate using commercially available ELISA kits (E‐EL‐H0149, Elabscience; E‐EL‐H0253c, Elabscience). Samples were assayed according to the manufacturer's guidelines. Each sample was subjected to three parallel experiments.

### 2.13 Statistical Analysis

The study conducted the statistical analysis using R and GraphPad Prism 7.0 (GraphPad Software Inc.). A one‐way ANOVA or Kruskal–Wallis test was utilized to compare three or more groups, with a significance set at *p* < 0.05. ROC curves were constructed to assess the pivotal genes' efficacy in diagnosing DN, and the AUC was calculated.

## 3 Results

### 3.1 DEGs Are Enriched in Inflammation Signaling Pathways in DN

**Figure 1:**
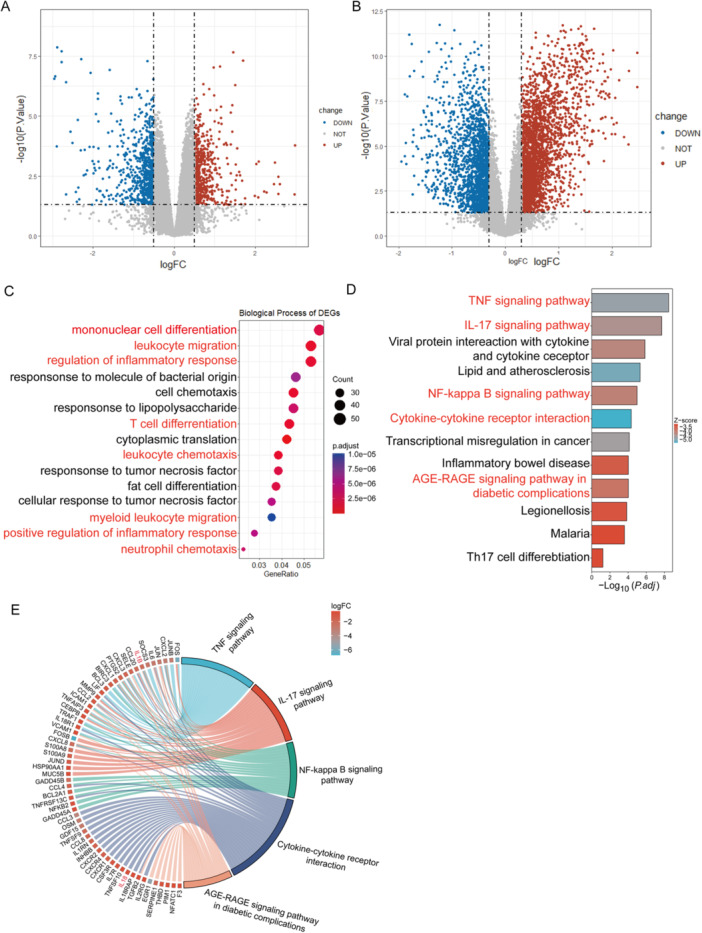
DEGs and their functional enrichment between the eDN and NC groups. Volcano plots showing DEGs between the eDN and NC groups in datasets GSE142025 (A) and GSE96804 (B). Blue indicates down‐regulated genes, red indicates the up‐regulated genes, and gray indicates the non‐significant genes. (C) Biological process enrichment of DEGs in dataset GSE142025. (D) KEGG pathway enrichment of DEGs in dataset GSE142025. (E) Chord plot illustrating DEGs enriched in the TNF signaling pathway, IL‐17 signaling pathway, NF‐κB signaling pathway, cytokine‐cytokine receptor interaction, and the AGE‐RAGE signaling pathway in diabetic nephropathy.

To investigate genetic changes in different stages of DN, we analyzed and identified DEGs in the GSE142025 and GSE96804 datasets [28]. The clinical characteristics of patients from two datasets were summarized in Table S1 based on previously published studies [18, 19, 20]. In the eDN versus NC groups, we found 580 up‐regulated and 689 down‐regulated genes in the GSE142025 dataset (*p* < 0.05 and |log FC| > 0.5), while 2953 up‐regulated and 1802 down‐regulated genes in the GSE96804 dataset (*p* < 0.05 and |log FC| > 0.3). The DEGs results for these datasets were presented in Figure 1A,B. The complete list of DEGs has been deposited in Figshare (DOI:10.6084/m9.figshare.30489911; https://figshare.com/). We subsequently conducted GO and KEGG enrichment analyses to investigate the function of these DEGs. GO analysis of the GSE142025 dataset revealed that the differential genes in eDN renal tissues were primarily involved in immune‐related biological processes such as monocyte, T cell differentiation, and leukocyte migration and chemotaxis, when compared with normal renal tissues (Figure 1C). The top 15 enriched GO terms in the eDN versus NC group and in the aDN versus the eDN group are shown in Tables S2 and S3, respectively. The KEGG analysis showed that the DEGs were mainly enriched in the IL‐17 signaling, TNF, and NF‐κB signaling pathways (Figure 1D,E). The KEGG pathway enrichment analysis of genes in the eDN versus NC group and in the aDN versus the eDN group is shown in Tables S4 and S5.

**Figure 2:**
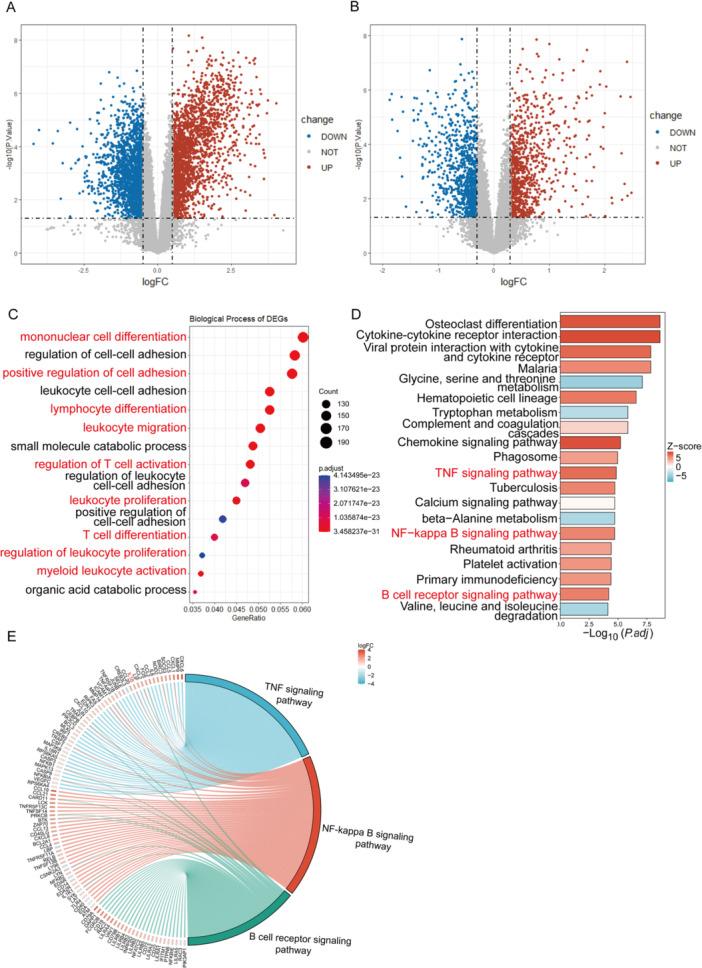
DEGs and their functional enrichment between aDN and eDN groups. Volcano plots showing DEGs between aDN and eDN groups in datasets GSE142025 (A) and GSE96804 (B). Blue indicates down‐regulated genes, red indicates the up‐regulated genes, and gray indicates the non‐significant genes. (C) Biological process enrichment of DEGs in dataset GSE142025. (D) KEGG pathway enrichment of DEGs in dataset GSE142025. (E) Chord plot illustrating DEGs enriched in the TNF signaling pathway, NF‐κB signaling pathway, and B cell receptor signaling pathway.

Similarly, differential gene expression analysis was performed between the aDN and eDN groups. Compared with the eDN group, the aDN group exhibited 2037 up‐regulated genes and 1834 down‐regulated genes in the GSE142025 dataset while displaying 950 up‐regulated genes and 934 down‐regulated genes in the GSE96804 dataset (Figure 2A,B). Subsequently, GO analysis of DEGs in the GSE142025 dataset was conducted. It revealed that the DEGs were also primarily enriched in biological processes such as monocyte and lymphocyte differentiation, migration and proliferation of leukocytes, and regulation of T cell activation (Figure 2C). Furthermore, KEGG pathway analysis indicated that the DEGs were mainly associated with the B‐cell receptor signaling pathway, TNF signaling pathway, and NF‐κB signaling pathway (Figure 2D,E).

### 3.2 Key Genes Are Screened by WGCNA in DN

**Figure 3:**
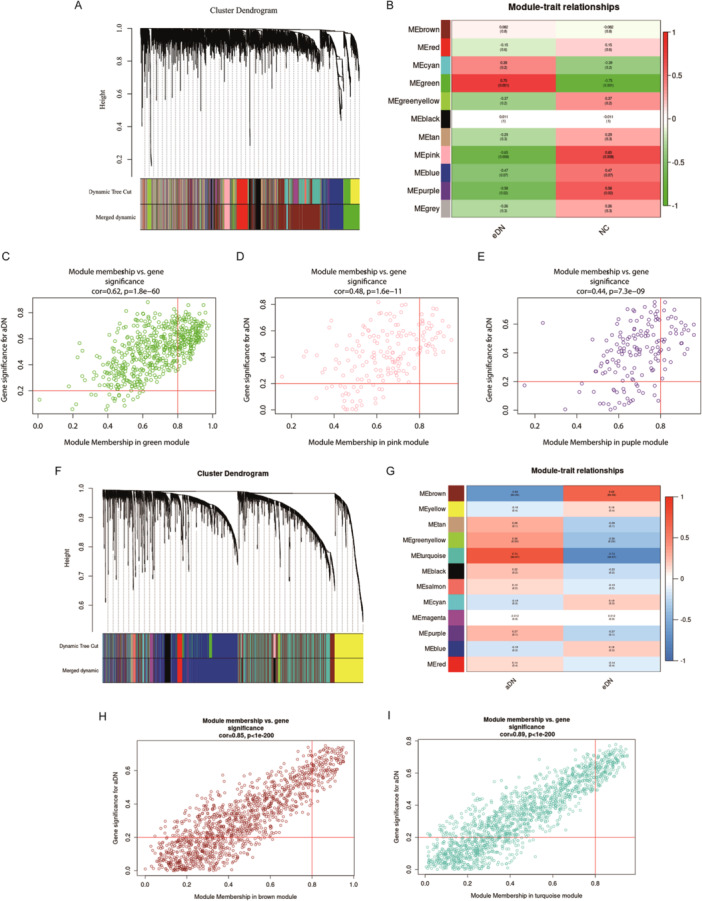
WGCNA co‐expression network analysis. (A) Gene expression clustering dendrogram of dataset GSE142025, including the eDN and NC groups. (B) Module‐phenotype correlation plots for the eDN and NC group. Each cell contains both the correlation coefficient and significance. (C–E) Scatter plots showing gene‐trait correlations for green, pink, and brown modules, respectively. The horizontal axis represents module membership (MM), the correlation between the module gene and the module. Higher MM values indicate a higher correlation between these genes and this module. The vertical axis represents gene significance (GS), the absolute correlation value between the module gene and the clinical phenotype. A higher GS value indicated a higher correlation between this gene and this phenotypic trait. (F) Gene expression clustering dendrogram for dataset GSE96804, comparing the aDN and eDN groups. (G) Module‐phenotype correlation plots for the aDN and eDN groups. (H, I) Scatter plots of gene‐trait correlation between brown (H) and blue modules (I), respectively.

Then, WGCNA was used to identify gene co‐expression modules associated with disease and key genes based on the correlation of gene expression profiles. Thus, we initially performed the WGCNA between the eDN and NC groups in GSE142025. The dendrogram (Figure 3A) and heat map (Figure 3B) of gene‐module correlations revealed that the green, pink, and purple modules were strongly associated with the occurrence of eDN. Accordingly, we visually screened genes within these three modules by plotting a scatterplot of GS versus module membership (MM) (Figure 3C–E), resulting in the selection of 227 key genes using GS > 0.2 and MM > 0.8 as selection criteria.

Likewise, we obtained a dendrogram of gene expression data from the aDN group compared to the eDN group (Figure 3F). A heat map of gene‐module correlations (Figure 3G) showed that the brown and turquoise modules were most strongly correlated with aDN. To select key genes, we plotted scatter plots of the brown and turquoise modules (Figure 3H,I), resulting in the selection of 312 key genes.

### 3.3 ZFP36, CLEC2D, and HCK Associated With NLRP3 Inflammasome Are Involved in the Progression of DN

**Figure 4:**
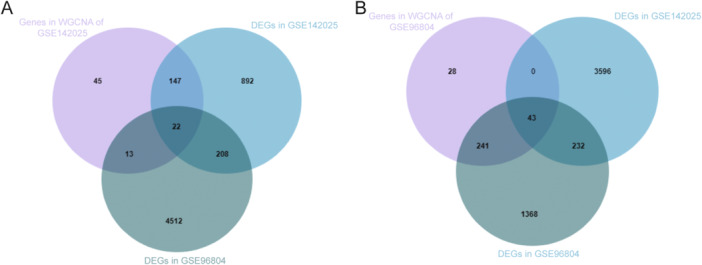
Venn diagrams depicting hub genes were generated using DEGs and key WGCNA genes from datasets GSE142025 and GSE96804. (A) Venn diagrams showing hub genes for eDN versus NC. (B) Venn diagrams showing hub genes for aDN versus eDN.

We then utilized the screened key genes and DEGs obtained from GSE142025 and GSE96804 to generate Venn diagrams (Figure 4A,B). Hub genes were acquired from the overlap. Compared with NC, we identified 22 hub genes (Table 1), including 4 upregulated genes (*FOXL1*, *GFRA2*, *MGC16275*, and *SDF2L1*) and 18 downregulated genes (*ATF3*, *BTG2*, *CCL3*, *CEP290*, *DUSP1*, *EVI2B*, *FOSB*, *HPGD*, *IKBIP*, *IL18*, *MNDA*, *NR4A3*, *PTGER3*, *PTGS2*, *S100A8*, *SNCA*, *XRCC4*, and *ZFP36*) in eDN. Compared with eDN, 43 hub genes were identified (Table 2), including 22 upregulated genes (*ABCA13*, *ARPC2*, *C3AR1*, *CD300E*, *CD44*, *CD86*, *CLEC2D*, *CSGALNACT2*, *DOCK8*, *E2F7*, *GIMAP2*, *HCK*, *IL4R*, *JAK1*, *LIF*, *MMD*, *PTPRN2*, *RAC2*, *SCIMP*, *SMOC2*, *SPARC* and *TEAD2*), and 18 downregulated genes (*ADAMTS17*, *ANKRD46*, *C1orf226*, *C1QL1*, *CACNA2D3*, *CALCR*, *DHRS4L1*, *FBXO24*, *KL*, *MIR4802*, *MPPED2*, *NAT8L*, *NYX*, *PAH*, *PPARA*, *PSAPL1*, *SLC5A11*, *TCF24*, *TGFBR3L*, *THNSL1*, and *TMEM178A*) in aDN. To better understand these hub genes, we conducted further literature research regarding them [29, 30, 31, 32, 33, 34]. We found that ZFP36, CLEC2D, and HCK have been reported to be associated with the activation of the NLRP3 inflammasome. We identified *ZFP36*, *CLEC2D*, and *HCK* as NLRP3 inflammasome‐associated hub genes (NIAHGs) (Table 3). Then, we focused on NIAHGs at different stages of DN. NIAHGs showed reliable diagnostic efficacy in the progression of DN.

**Table 1:** Hub genes from Figure 4A.

DEGs	Gene symbol
Upregulated DEGs	FOXL1; GFRA2; MGC16275; SDF2L1.
Downregulated DEGs	ATF3; BTG2; CCL3; CEP290; DUSP1; EVI2B; FOSB; HPGD; IKBIP; IL18; MNDA; NR4A3; PTGER3; PTGS2; S100A8; SNCA; XRCC4; **ZFP36**.

**Table 2:** Hub genes from Figure 4B.

DEGs	Gene symbol
Upregulated DEGs	ABCA13; ARPC2; C3AR1; CD300E; CD44; CD86; **CLEC2D**; RAC2; CSGALNACT2; DOCK8; E2F7; GIMAP2; **HCK**; IL4R; JAK1; LIF; MMD; PTPRN2; SCIMP; SMOC2; SPARC; TEAD2.
Downregulated DEGs	ADAMTS17; ANKRD46; C1orf226; C1QL1; CACNA2D3; CALCR; DHRS4L1; FBXO24; KL; MIR4802; MPPED2; NAT8L; PAH; PPARA; PSAPL1; SLC5A11; TCF24; TGFBR3L; THNSL1; NYX; TMEM178A.

**Table 3:** The description of NIAHGs.

Gene symbol	Official full name	Description
ZFP36	ZFP36 Ring Finger Protein	Involved in several processes, including cellular response to cytokine stimulus, cellular response to growth factor stimulus, and regulation of gene expression [67, 68, 69]. Acts upstream of or within the mRNA catabolic process [29, 30].
CLEC2D	C‐Type Lectin Domain Family 2 Member D	It encodes a member of the natural killer cell receptor C‐type lectin family [70, 71].
HCK	Hemopoietic Cell Kinase	The protein encoded by this gene is a member of the Src family of tyrosine kinases [33]. It may play a role in neutrophil migration and in the degranulation of neutrophils [72, 73].

**Figure 5:**
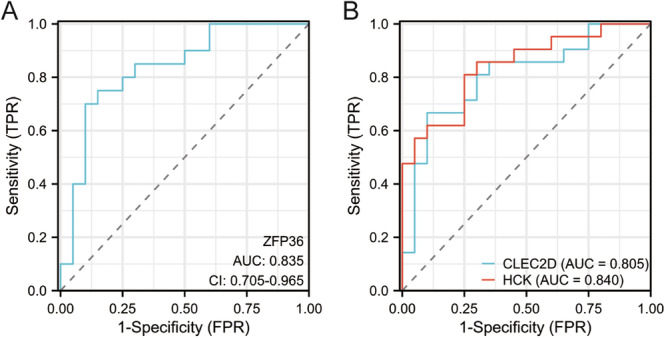
ROC analysis of NIAHGs. (A) The ROC curve analyzed the diagnostic efficacy of *ZFP36* for eDN versus NC. (B) ROC curve analyzed the diagnostic efficacy of *CLEC2D* and *HCK* for aDN versus eDN.

To identify the diagnostic value of NIAHGs, we performed ROC analysis in GSE142025. The result showed the AUC values for *ZFP36*, *CLEC2D*, and *HCK* were 0.835, 0.805, and 0.840, respectively (Figure 5A,B), suggesting the reliable predictive specificity and sensitivity performance of the genes in the progression of DN.

### 3.4 Validation of NIAHGs Expression Across Different Clinical Stages of DN

**Figure 6:**
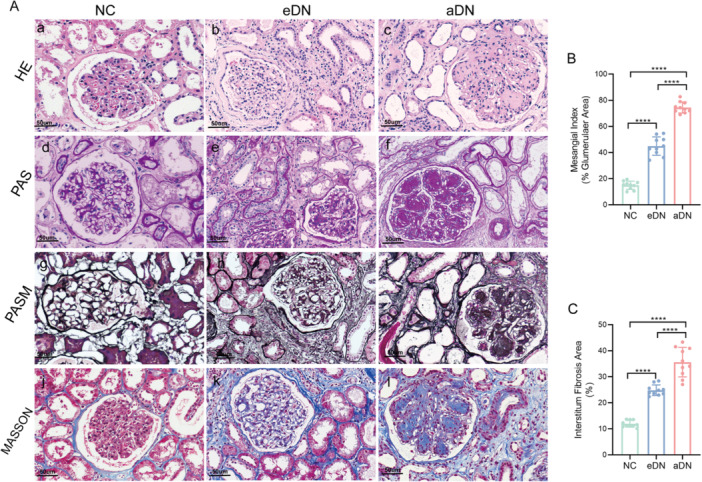
Histological changes in different stages of DN. (A) Representative HE, PAS, and Masson staining images (magnification: ×400) illustrate the histopathological changes in NC, eDN, and aDN samples. (B) Glomerular mesangial matrix expansion quantified from PAS staining (10 slides from 6 patients per group). (C) Quantitative assessment of the interstitial fibrosis area from Masson staining (10 slides from 6 patients per group). Values shown are means ± SD. ****p < 0.0001. Significance was assessed by one‐way ANOVA followed by Tukey's post hoc test.

To reveal the pathological changes in different clinical stages of DN, we performed HE, PAS, PASM, and Masson staining on renal tissues from 18 patients with three groups (Figure 6A–C). Based on the HE staining results, we observed that the interstitium of patients with DN showed significant inflammatory cell infiltration compared with normal renal tissue (Figure 6A). PAS and PASM staining showed that, with the continuous progression of the disease, the glomerular basement membrane gradually thickened, and mesangial matrix progressively accumulated (Figure 6B), which eventually developed into nodular glomerulosclerosis, forming Kimmelstiel–Wilson (K–W) nodules. At the same time, the basement membranes of the renal tubules showed a tendency to thicken and wrinkle. The results of Masson staining showed that, especially in aDN, atrophic renal tubules appeared, and the interstitium was significantly dilated, accompanied by signs of fibrosis (Figure 6C).

**Figure 7:**
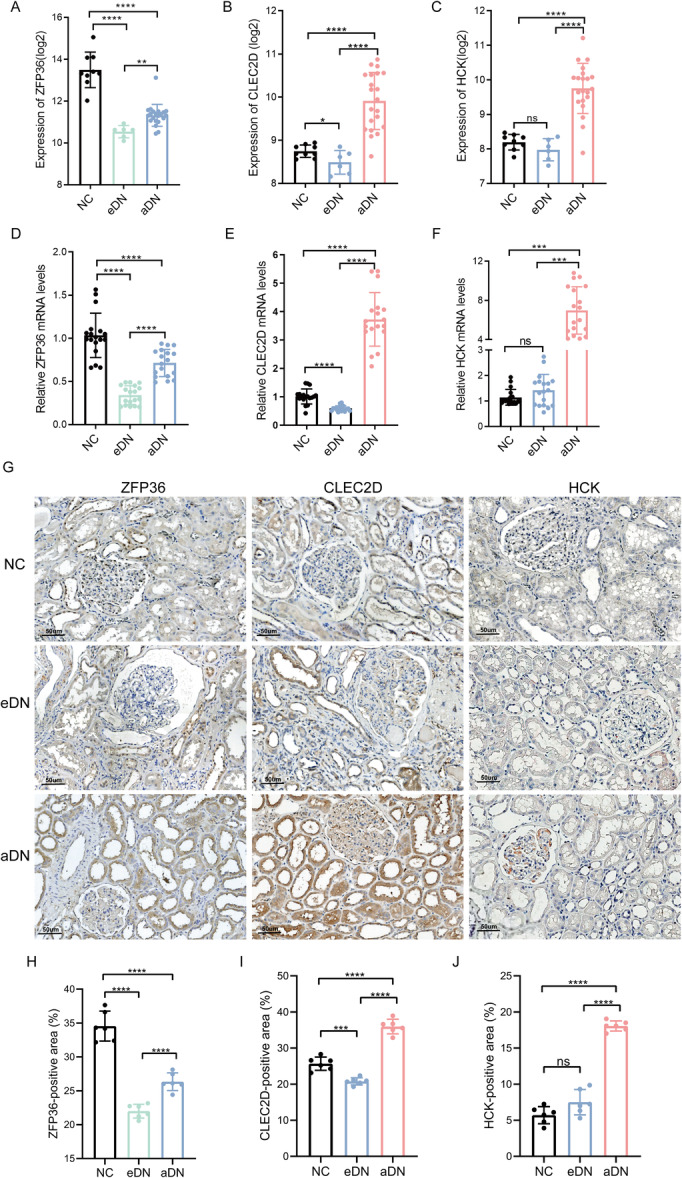
Validation of the expression levels of NIAHGs. (A–C) The expression of *ZFP36* (A), *CLEC2D* (B), and *HCK* (C) in the GSE142025 dataset. (D–F) Validation of the expression of *ZFP36* (D), *CLEC2D* (E), and *HCK* (F) in renal tissues from DN patients through RT‐qPCR (*n* = 6, three technical replicates). (G) The protein expression of ZFP36, CLEC2D, and HCK in renal tissues from DN patients through IHC staining (magnification: ×400, *n* = 6). (H–J) Quantitative analysis of the positive staining area of ZFP36 (H), CLEC2D (I), and HCK (J) in renal tissues of NC, eDN, and aDN groups, respectively. Scale bars = 50 μm. Data are mean ± SD. *p < 0.05, **p < 0.01, ***p < 0.001, and ****p < 0.0001. Significance determined by one‐way ANOVA followed by Tukey's post hoc test.

To further investigate NIAHGs, we first visualized their expression in the dataset GSE142025 using scatter plots. Compared to the NC group, *ZFP36* expression exhibited a significant decrease in both the early and advanced stages of DN, with a notable increase observed in the advanced stage relative to the early stages (Figure 7A). Similarly, *CLEC2D* was reduced in eDN but rose markedly in the advanced stage (Figure 7B). On the other hand, *HCK* displayed no significant changes between NC and eDN but manifested a more pronounced increase in aDN than in NC and eDN (Figure 7C).

**Figure 8:**
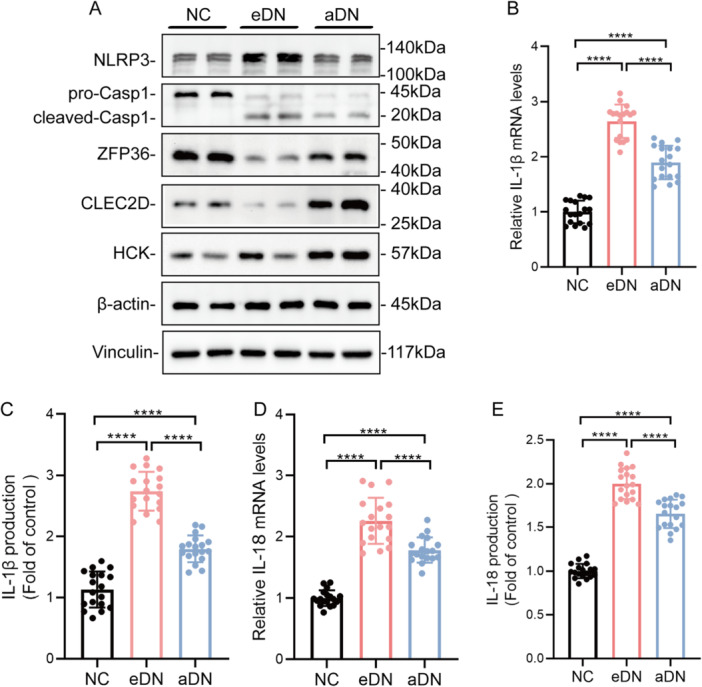
Expression of molecules related to the activation of NLRP3 inflammasomes and NIAHGs. (A) Immunoblot analysis of NLRP3, pro‐caspase‐1, caspase‐1 p20, and NIAHGs proteins in renal tissues from NC, eDN, and aDN patients. (B–E) Relative IL‐1β and IL‐18 levels across groups determined by RT‐qPCR (B, C) and ELISA (D, E). *n* = 6, three technical replicates. Data are mean ± SD. ****p < 0.0001. Significance determined by one‐way ANOVA followed by Tukey's post hoc test.

Subsequently, we confirmed the results above by qRT‐PCR (Figure 7D–F), IHC (Figure 7G), and Western blot (Figure 8A). Indeed, the trend of NIAHGs expression was consistent with the conclusion in GSE142025.

### 3.5 Immunoblot and ELISA Analyses Reveal the NIAHGs Expression Is Associated With the Activation of NLRP3 Inflammasome

To better evaluate the association between NIAHGs and NLRP3 inflammasome activation at different stages of DN, we utilized immunoblot, qRT‐PCR, and ELISA analyses to identify whether NIAHGs expression is associated with the activation of NLRP3 inflammasome. The results showed that the expression of NIAHGs was negatively correlated with the activation of the NLRP3 inflammasome. Specifically, the NLRP3 inflammasome exhibited substantial activation in eDN and subsequently underwent attenuation in aDN. Conversely, the expression levels of NIAHGs were low in eDN and showed an increase in aDN (Figure 8A). Furthermore, both mRNA and protein levels of IL‐1β and IL‐18 were significantly elevated during the eDN phase, which strongly suggested NLRP3 inflammasome activation (Figure 8B–E). Therefore, the NLRP3 inflammasome was significantly activated, leading to the release of large amounts of IL‐1β and IL‐18 in eDN, further initiating the inflammatory response in the diabetic kidney.

## 4 Discussion

Due to the rapidly increasing prevalence of diabetes in recent decades, DN has become the leading cause of ESRD [35]. Unfortunately, the lack of specific and effective therapeutic interventions impedes the clinical management of DN. It is primarily due to an incomplete understanding of DN pathogenesis. Thus, there is an urgent need to identify new biomarkers for DN progression. To address the problem, we analyzed microarray datasets from 98 samples across GSE142025 and GSE96804, including normal and DN tissue. Through analysis of DEGs, WGCNA, and an in‐depth literature survey, we identified three hub genes as NIAHGs (*ZFP36*, *CLEC2D*, *HCK*) and revealed that the NIAHGs expression is associated with the activation of NLRP3 inflammasome in DN progression. Our findings reveal critical gene associations that could serve as potential biomarkers of disease progression, thereby improving diagnostic accuracy and informing therapeutic strategies. The integration of clinical validation further strengthens the translational relevance of this research and provides a foundation for future studies aimed at mitigating the impact of DN on patient health and healthcare systems.

GO enrichment analysis of DEGs indicated the active participation of immune cells, such as T cells and monocytes, in the progression of DN at both early and advanced stages. This discovery can be attributed to the persistent secretion of inflammatory factors in DN patients, induced by chronic hyperglycemia, hyperlipidemia, and other hemodynamic and metabolic abnormalities in diabetic individuals, leading to a cascade of inflammatory reactions subsequently [36]. In the last decade, various cellular and molecular investigations have consistently demonstrated that inflammation plays a crucial role in the pathophysiology of DN [37]. Meanwhile, the KEGG results showed that the NF‐κB signaling pathway was involved throughout the disease development. NF‐κB plays an essential role in a series of biological processes, such as promoting macrophage infiltration and inducing activation of transforming growth factor β (TGF‐β), thereby promoting extracellular matrix accumulation and renal fibrosis, and even inducing damage to podocytes [38, 39]. The NF‐κB pathway exerts critical regulatory control over multiple downstream molecules, including members of the inflammasome family, with a particular emphasis on the NLRP3 inflammasome. This inflammasome assumes a central role in the inflammatory immune responses and functions by promoting the production and maturation of pro‐inflammatory factors [40, 41]. Furthermore, mounting evidence suggests a close association between the activation of NLRP3 inflammasome and fibrosis, indicating the potential significance of this inflammasome in tissue remodeling and repair processes [42]. Numerous investigations have substantiated a surge in both the expression of NLRP3 mRNA and the activation of NLRP3 inflammasome in the kidneys of individuals with type 2 diabetes [43, 44].

The NLRP3 inflammasome is a macromolecular complex with a molecular weight of around 700 kDa, comprising NOD‐like receptor protein 3 (NLRP3), cysteine aspartate protease 1 (caspase‐1), and apoptosis‐associated speck‐like protein containing a CARD (ASC) [45]. In the human kidney, the NLRP3 inflammasome undergoes activation primarily in immune cells, including macrophages and dendritic cells. However, it is also expressed in renal cells such as podocytes, mesangial cells, and glomerular endothelial cells [46, 47, 48, 49].

In the context of NLRP3 inflammasome activation, the first step involves the activation of the nuclear transcription factor NF‐κB, which responds to multiple pathological signals. This activation, in turn, promotes the transcription and expression of several key molecules, including NLRP3, ASC, caspase‐1 precursor, IL‐1β precursor, and IL‐18 precursor. Subsequently, the assembly of the NLRP3 inflammasome occurs through multiple activation pathways, which include K^+^ channel opening, lysosomal disruption, and aberrant accumulation of ROS in the classical activation mode [50, 51, 52]. Following the NLRP3 inflammasome activation, caspase‐1 precursor is activated, leading to the cleavage of IL‐1β precursor and IL‐18 precursor to generate the proinflammatory and mature cytokines IL‐1β and IL‐18 [53, 54]. These activated cytokines play critical roles in mediating the inflammatory response. The effects of IL‐1β and IL‐18 on the kidney result in glomerular damage through various mechanisms, including increased endothelial cell permeability, alteration of glomerular hemodynamics, disruption of prostaglandin synthesis, the stimulation of mesangial cells and fibroblast proliferation, and induction of TGF‐β production [55, 56, 57]. These inflammatory factors act synergistically, resulting in renal tubular interstitial fibrosis and decreased glomerular filtration rate, ultimately leading to renal injury. Concurrently, in patients with DN, glucose tolerance and insulin sensitivity are further impaired [58, 59]. In the present study, we consistently provide a detailed analysis of NLRP3 inflammasome activation in different stages of DN patients. Indeed, it is accompanied by elevated expressed NLRP3 protein level, cleaved‐caspase‐1 (p20), and increased secretion of IL‐1β.

ZFP36 directly binds to the 3′‐untranslated region of NLRP3 mRNA, leading to its targeted degradation. Moreover, ZFP36 hinders the translation and activation of IL‐1β. Thus, ZFP36 efficiently suppresses the activation of NLRP3 inflammasome, which plays a negative role in disease progression [30]. Likewise, our results showed that the expression of ZFP36 inversely correlated with NLRP3 inflammasome activation in DN tissues. Previous studies have revealed that ZFP36 expression is downregulated in DN, leading to the upregulation of interleukin‐17 (IL‐17) and claudin‐1 expression. This molecular dysregulation ultimately results in podocyte injury, a characteristic feature of DN pathogenesis [60]. In addition, prolonged stress triggers microglia activation in the medial prefrontal cortex (mPFC) by stimulating extracellular nucleosomes via CLEC2D and TLR9. This activation leads to the production of ROS, the initiation of the NF‐κB signaling pathway, and the activation of NLRP3 inflammasome [32]. We also found the expression of CLEC2D inversely correlated with NLRP3 inflammasome activation in eDN renal tissues. We observed that HCK showed no significant changes in both the NC and eDN, but manifested a more pronounced increase in aDN than in NC and eDN. Notably, the HCK kinase activity inhibitor A419259 effectively suppresses macrophage and NLRP3 inflammasome activity in microglia and attenuates lipopolysaccharide‐induced inflammatory responses in vivo [34]. This observation differs slightly from our experimental results, possibly due to sample size, subject variability, and disease type.

In the present study, we also observed several diabetes‐related DEGs that, while not central to our core findings, warrant further discussion. A notable example is PEA15 (Phosphoprotein Enriched in Diabetes/Astrocytes 15), a multifunctional scaffold protein renowned for its critical roles in regulating insulin signaling, apoptosis, and autophagy [61, 62]. Its significance in diabetes is well‐established, with prior studies linking its overexpression to systemic insulin resistance and its involvement in complications such as diabetic kidney disease [63, 64]. The identification of PEA15 as a DEG in our cohort independently corroborates these previous findings and reinforces its consistent association with the diabetic milieu. However, PEA15 was not identified as a hub gene in our subsequent WGCNA and functional enrichment analyses. We postulate that its primary role lies in shaping the underlying pathogenic environment—for instance, by promoting insulin resistance and metabolic dysregulation or by modulating autophagic flux and cellular survival. These processes can create a permissive state that facilitates the activation of the core inflammatory cascade driven by the hub genes we identified (*ZFP36*, *CLEC2D*, and *HCK*), without PEA15 itself being a direct, integral component of the inflammasome complex.

DN is traditionally characterized by progressive proteinuria. However, emerging evidence indicates that DN can also progress through non‐proteinuric pathways, with up to 20% of patients experiencing a decline in renal function without significant proteinuria [65]. These non‐proteinuric progression patterns may be driven by alternative mechanisms, including glomerular endothelial dysfunction, tubulointerstitial injury, renal fibrosis, inflammation, oxidative stress, and dysregulation of apoptosis or autophagy. These pathways underscore the complexity of DN progression and challenge the reliance on proteinuria as the sole indicator of disease severity. The existence of non‐proteinuric progression patterns has important implications for our study. Our findings are based on a cohort of patients with proteinuric DN, and the limited sample size may restrict the generalizability of our conclusions to patients with non‐proteinuric disease.

The histopathology of DN exhibits distinct characteristics at various stages. Early‐stage DN is typically characterized by the glomerular basement membrane thickening and mesangial expansion, while advanced‐stage DN often presents with significant glomerular sclerosis and renal interstitial fibrosis, with K–W nodules representing a characteristic histological hallmark. Our pathological images align with this spectrum. However, K–W nodules are not universally present, and their occurrence is heterogeneous and focal. Viggiano et al. found that K–W nodules are more common in type 1 diabetes than in type 2, suggesting that this difference is not solely related to hyperglycemia but may be influenced by different pathogenic factors, including insulin signaling, C‐peptide levels, and dyslipidemia [66]. Therefore, the absence of obvious K–W nodules in certain advanced cases reflects the diversity of underlying molecular drivers and the complex interplay of metabolic and non‐glycemic pathways in disease progression.

This study has several limitations that should be acknowledged. First, we acknowledge that the current staging framework for DN, which relies on conventional clinical parameters such as proteinuria and eGFR, has inherent methodological limitations. Patients without proteinuria were not included. These parameters may not adequately capture the heterogeneity of DN subgroups. Second, while our findings highlight significant associations between NLRP3 inflammasome activation and the progression of DN, the causal relationship remains unconfirmed. Although existing evidence from preclinical studies suggests that NLRP3 inhibitors can attenuate renal inflammation and injury, our current study lacks functional validation to definitively establish causality. Similarly, our study suggested a clear association between NIAHG expression and NLRP3 inflammasome activation, but lacked functional assays to confirm the causal relationship between NIAHGs and NLRP3. Third, clinical translation is further constrained by unadjusted confounders such as glycemic fluctuations, antihypertensive therapies, and comorbidities (cardiovascular disease), which may influence biomarker specificity in real‐world settings. Fourth, the clinical value of biomarkers for the diagnosis and prognosis of DN requires validation through long‐term follow‐up, prospective cohort studies, and robust real‐world evidence. Therefore, we emphasize that the findings of this study should be regarded as exploratory results rather than causal confirmation. These findings warrant validation in future well‐designed, prospective, longitudinal studies. Finally, the verification was conducted with a smaller sample size. In the future, it is necessary to carry out the verification with a larger sample size.

To address these gaps, future work should prioritize: (1) refining DN stratification by integrating histopathological data with multi‐omics profiling to identify subtype‐specific biomarkers; (2) elucidating causal mechanisms through interventions in transgenic animal models and patient‐derived kidney cells; and (3) prospective clinical validation in multicenter cohorts with standardized protocols to assess the diagnostic and prognostic utility of these biomarkers while accounting for confounding factors. In addition, combining transcriptomic data with single‐cell transcriptomic analysis and spatial transcriptomic analysis may uncover novel pathways linking NLRP3 activation to DN progression and ultimately guide targeted therapeutic strategies.

Taken together, this study identified the hub genes in DN progression using two microarray datasets and integrated bioinformatics analysis. We used human renal samples for cross‐validation and supported our findings based on bioinformatics analysis. Importantly, we revealed that NIAHGs expression was associated with the activation of the NLRP3 inflammasome. Moreover, the NLRP3 inflammasome was significantly activated, leading to the release of large amounts of IL‐1β and IL‐18 in eDN, further initiating the inflammatory response in the diabetic kidney. This study reveals the potential role of NIAHG in DN progression, providing a theoretical basis and candidate targets for subsequent in‐depth mechanistic research and translational exploration.

## Author Contributions

**Sheng Zhao:** conceptualization (equal), investigation (equal), formal analysis (equal), writing – original draft (lead). **Yuejiao Li:** investigation (equal), formal analysis (equal), writing – review and editing (equal). **Wenchuan Li:** investigation (equal), formal analysis (equal). **Lan Dong:** data curation (equal), writing – original draft (supporting). **Rong Lian:** data curation (equal), writing – original draft (supporting). **Qingqing Luo:** visualization (equal). **Xingji Lian:** visualization (equal). **Jianbo Li:** writing – review and editing (equal). **Feng He:** conceptualization, formal analysis (equal), writing – review and editing (equal).

## Ethics Statement

The study involving human participants was performed in accordance with the 1964 Helsinki Declaration and its later amendments, and approved by the Ethics Committee of Guangzhou First People's Hospital (approval No. K‐2022‐014‐01).

## Conflicts of Interest

The authors declare no conflicts of interest.

## Supporting information


**Supplementary Figure 1.** Determination of the soft threshold power of the WGCNA in the eDN and NC group. **Supplementary Figure 2.** Determination of the soft threshold power of the WGCNA in the aDN and eDN group. **Supplementary Figure 3.** Representative immunohistochemical images of ZFP36, CLEC2D, and HCK in renal tissues from DN patients at different magnifications. **Supplementary Figure 4.** Immunohistochemical staining of ZFP36, CLEC2D, and HCK in renal tissues from DN patients across different microscopic fields. **Supplementary Figure 5.** Original Western blot images and annotations. **Supplementary Table 1.** Demographic and clinical characteristics of kidney biopsy patients in GSE142025 and GSE96804. **Supplementary Table 2.** The top 15 GO enrichment terms of genes in the eDN versus the NC group. **Supplementary Table 3.** The top 15 GO enrichment terms of genes in the aDN versus the eDN group. **Supplementary Table 4.** The KEGG pathway enrichment analysis of genes in the aDN versus the eDN group. **Supplementary Table 5.** The KEGG pathway enrichment analysis of genes in the eDN versus the NC group. **Supplementary Table 6.** Baseline characteristics of DN patients and the control group in the validation cohort.

## Data Availability

The datasets presented in this study can be found in online repositories: GSE142025 (https://www.ncbi.nlm.nih.gov/geo/query/acc.cgi?acc=GSE142025) and GSE96804 (https://www.ncbi.nlm.nih.gov/geo/query/acc.cgi?acc=GSE96804).
