# Supplementary material for: Characterization of NLRP3 Inflammasome‐Associated Hub Genes in the Progression of Diabetic Nephropathy
Source: Immun Inflamm Dis. 2026 Apr 13;14(4):e70424. doi: 10.1002/iid3.70424 (PMC13076925; doi:10.1002/iid3.70424)
Supplement: Supplementary file 1 — Supplementary Figure 1. Determination of the soft threshold power of the WGCNA in the eDN and NC group. Supplementary Figure 2. Determination of the soft threshold power of the WGCNA in the aDN and eDN group. Supplementary Figure 3. Representative immunohistochemical images of ZFP36, CLEC2D, and HCK in renal tissues from DN patients at different magnifications. Supplementary Figure 4. Immunohistochemical staining of ZFP36, CLEC2D, and HCK in renal tissues from DN patients across different microscopic fields. Supplementary Figure 5. Original Western blot images and annotations. Supplementary Table 1. Demographic and clinical characteristics of kidney biopsy patients in GSE142025 and GSE96804. Supplementary Table 2. The top 15 GO enrichment terms of genes in the eDN versus the NC group. Supplementary Table 3. The top 15 GO enrichment terms of genes in the aDN versus the eDN group. Supplementary Table 4. The KEGG pathway enrichment analysis of genes in the aDN versus the eDN group. Supplementary Table 5. The KEGG pathway enrichment analysis of genes in the eDN versus the NC group. Supplementary Table 6. Baseline characteristics of DN patients and the control group in the validation cohort. [file IID3-14-e70424-s001.pdf]

Legends of Supplement Figure and Table

Supplementary Table 1. Demographic and clinical characteristics of kidney biopsy patients in GSE142025 and GSE96804.

Supplementary Table 2. The top 15 GO enrichment terms of genes in the eDN versus the NC group.

Supplementary Table 3. The top 15 GO enrichment terms of genes in the aDN versus the eDN group.

Supplementary Table 4. The KEGG pathway enrichment analysis of genes in the aDN versus the eDN group.

Supplementary Table 5. The KEGG pathway enrichment analysis of genes in the eDN vers us the NC group.

Supplementary Table 6 . Baseline characteristics of DN patients and the control group in the validation cohort.

Supplementary Figure 1. Determination of the soft threshold power of the WGCNA in the eDN and NC group.

Supplementary Figure 2. Determination of the soft threshold power of the WGCNA in the aDN and eDN group.

Supplementary Figure 3. Representative immunohistochemical images of ZFP36, CLEC2D, and HCK in renal tissues from DN patients at different magnifications.

Supplementary Figure 4. Immunohistochemical staining of ZFP36, CLEC2D, and HCK in renal tissues from DN patients across different microscopic fields.

Supplementary Figure 5. Original Westernblot images and annotations.

Supplementary Table 1. Demographic and clinical characteristics of kidney biopsy patients in GSE142025 and GSE96804.

Baseline characteristics	Control	eDN	aDN	P
**GSE142025**	(n = 9)	(n = 6)	(n = 22)	
Age (years)	60.89 ± 2.85	50.67 ± 3.547	53.18 ± 2.467	0.6261
BMI (kg/m^2)	23.38 ± 1.39	24.68 ± 1.243	25.57 ± 0.697	0.5523
eGFR (ml/min/1.73m^2)	87.33 ± 9.84	117.7 ± 8.624	63.79 ± 5.765	0.0001
Proteinuria (g/24h)	0.09 ± 0.01	0.22 ± 0.038	6.11 ± 0.967	0.0042
UACR (mg/g)	16.88 ± 0.69	130.2 ± 23.68	4444 ± 674.1	0.0028
Global glomerulosclerosis (%)	NA	2.381 ± 0.048	21.603 ± 0.187	0.0005
**GSE96804**	(n = 20)	(n = 20)	(n = 21)	
Age (years)	43.2 ± 6.5	46.5 ± 8.7	47.7± 7.0	0.835
BMI (kg/m2)	21.6 ± 2.52	25.2 ± 2.12	24.6 ± 1.22	0.514
eGFR (ml/min/1.73m2)	100.23 ± 10.5	99.16 ± 15.47	42.61 ± 13.47	<0.001
Proteinuria (g/24h)	NA	0.60 ± 0.27	5.41 ± 3.88	<0.001
Glomerular lesions, n				<0.001
Class I	NA	5	0	
Class II	NA	15	0	
Class III	NA	0	16	
Class IV	NA	0	5	

Data are presented as n or mean ± SD. The two-sided unpaired t-test was used to determine the statistical significance between eDN and aDN. NA, Not available; eDN, early diabetic nephropathy; aDN, advanced diabetic nephropathy; BMI, body mass index; eGFR, estimated glomerular filtration rate; UACR, urinary albumin-to-creatinine ratio.

Supplementary Table 2. The top 15 GO enrichment terms of genes in the eDN versus the NC group.

ID	Description	GeneRatio	pvalue	Count
**BP**				
**GO:1903131**	mononuclear cell differentiation	48/574	1.01119E-14	48
**GO:0050900**	leukocyte migration	41/574	3.27764E-12	41
**GO:0050727**	regulation of inflammatory response	40/574	2.93576E-11	40
**GO:0002237**	response to molecule of bacterial origin	42/574	4.95429E-14	42
**GO:0060326**	cell chemotaxis	38/574	4.89383E-13	38
**GO:0032496**	response to lipopolysaccharide	41/574	2.84285E-14	41
**GO:0030217**	T cell differentiation	37/574	7.90915E-15	37
**GO:0002181**	Cytoplasmic translation	35/574	9.86343E-10	35
**GO:0030595**	leukocyte chemotaxis	33/574	2.76515E-13	33
**GO:0034612**	response to tumor necrosis factor	27/574	1.26416E-08	27
**GO:0045444**	Fat cell differentiation	28/574	5.39612E-05	28
**GO:0071219**	cellular response to molecule of bacterial origin	26/574	9.03919E-09	26
**GO:0097529**	myeloid leukocyte migration	30/574	1.8339E-11	30
**GO:0050729**	positive regulation of inflammatory response	22/574	5.50531E-10	22
**GO:0042119**	neutrophil activation	5/574	0.007886358	5

Supplementary Table 3. The top 15 GO enrichment terms of genes in the aDN versus the eDN group.

ID	Description	GeneRatio	pvalue	Count
**BP**				
**GO:1903131**	mononuclear cell differentiation	183/3174	2.17208E-36	183
**GO:0022407**	regulation of cell-cell adhesion	179/3174	9.77525E-31	179
**GO:0045785**	positive regulation of cell adhesion	174/3174	1.59739E-29	174
**GO:0007159**	leukocyte cell-cell adhesion	161/3174	4.16466E-32	161
**GO:0030098**	lymphocyte differentiation	161/3174	6.03104E-32	161
**GO:0050900**	leukocyte migration	154/3174	9.25638E-28	154
**GO:0044282**	small molecule catabolic process	150/3174	8.8095E-27	150
**GO:0050863**	regulation of T cell activation	146/3174	8.85744E-30	146
**GO:1903037**	regulation of leukocyte cell-cell adhesion	143/3174	9.57874E-28	143
**GO:0070661**	leukocyte proliferation	137/3174	1.4644E-26	137
**GO:0022409**	positive regulation of cell-cell adhesion	128/3174	9.70475E-28	128
**GO:0030217**	T cell differentiation	121/3174	1.75232E-28	121
**GO:0070663**	regulation of leukocyte proliferation	113/3174	3.84603E-25	113
**GO:0002274**	myeloid leukocyte activation	114/3174	4.26938E-30	114
**GO:0016054**	organic acid catabolic process	108/3174	3.20173E-24	108

Supplementary Table 4. The KEGG pathway enrichment analysis of genes in the eDN versus the NC group.

ID	Description	p.adjust	zscore
**hsa04668**	TNF signaling pathway	3.38719E-09	-4.795831523
**hsa04657**	IL-17 signaling pathway	1.97384E-08	-4.472135955
**hsa04061**	Viral protein interaction with cytokine and cytokine receptor	1.41027E-06	-4.242640687
**hsa05417**	Lipid and atherosclerosis	4.69226E-06	-5.099019514
**hsa04064**	NF-kappa B signaling pathway	1.0186E-05	-4.123105626
**hsa04060**	Cytokine-cytokine receptor interaction	4.52433E-05	-5.385164807
**hsa05202**	Transcriptional misregulation in cancer	7.35125E-05	-4.69041576
**hsa05321**	Inflammatory bowel disease	9.41236E-05	-3.464101615
**hsa04933**	AGE-RAGE signaling pathway in diabetic complications	9.41236E-05	-3.872983346
**hsa05134**	Legionellosis	0.000140691	-3.31662479
**hsa05144**	Malaria	0.00024816	-3.16227766
**hsa04659**	Th17 cell differentiation	0.059556508	-3.16227766

Supplementary Table 5. The KEGG pathway enrichment analysis of genes in the aDN versus the eDN group.

ID	Description	p.adjust	zscore
**hsa04380**	Osteoclast differentiation	2.26109E-09	7.487767803
**hsa04060**	Cytokine-cytokine receptor interaction	2.26109E-09	8.390470785
**hsa04061**	Viral protein interaction with cytokine and cytokine receptor	1.43848E-08	6.714285714
**hsa00260**	Glycine, serine and threonine metabolism	7.82485E-08	-4.706787243
**hsa04640**	Hematopoietic cell lineage	2.62857E-07	5.897678246
**hsa00380**	Tryptophan metabolism	1.39556E-06	-3.4
**hsa04610**	Complement and coagulation cascades	1.39556E-06	1.897366596
**hsa04062**	Chemokine signaling pathway	6.01789E-06	7.6494631
**hsa04145**	Phagosome	1.074E-05	4.201805852
**hsa04668**	TNF signaling pathway	1.40596E-05	6.487446071
**hsa05152**	Tuberculosis	1.85028E-05	5.333493587
**hsa04020**	Calcium signaling pathway	1.92865E-05	0.333333333
**hsa00410**	beta-Alanine metabolism	1.92865E-05	-3.900067476
**hsa04064**	NF-kappa B signaling pathway	1.95424E-05	5.337449962
**hsa05323**	Rheumatoid arthritis	3.82633E-05	4.003203845
**hsa05340**	Primary immunodeficiency	4.04961E-05	4.582575695
**hsa04662**	B cell receptor signaling pathway	6.33887E-05	5.578018081
**hsa00280**	Valine, leucine and isoleucine degradation	7.45306E-05	-4.082482905

Supplementary Table 6. Baseline characteristics of D N patients and controls in the validation cohort.

Group	Control (n = 6)	eDN (n = 6)	aDN (n=6)
Sex (M/F)	4/2	2/4	3/3
Age (yr)	59 ± 12.6	52 ± 6.7	54 ± 9.3
BMI (kg/m^2)	22.3 ± 0.6	25.6 ± 1.6	29.2 ± 0.7
DM Duration (yr)	0	5.0 ± 0.8	11.0 ± 2.1
Serum creatinine (μmol/L)	82 ± 10.90	69 ± 11.66	255 ± 121.29
UACR (mg/g)	10 ± 1.88	182.5 ± 119.90	1104 ± 634.82
HbA1c (%)	5.4 ± 0.1	6.9 ± 0.2	9.1 ± 0.4
Hemoglobin (g/L)	137 ± 1.87	127 ± 2.16	103 ± 9.35
eGFR (mL/min/1.73m^2)	83 ± 16.41	100 ± 7.41	32 ± 25.76

Abbreviation: BMI, body mass index; DM, diabetes mellitus; UACR, urinary albumin-to-creatinine ratio; HbA1c, glycated hemoglobin A1c; eGFR, estimated glomerular filtration rate.

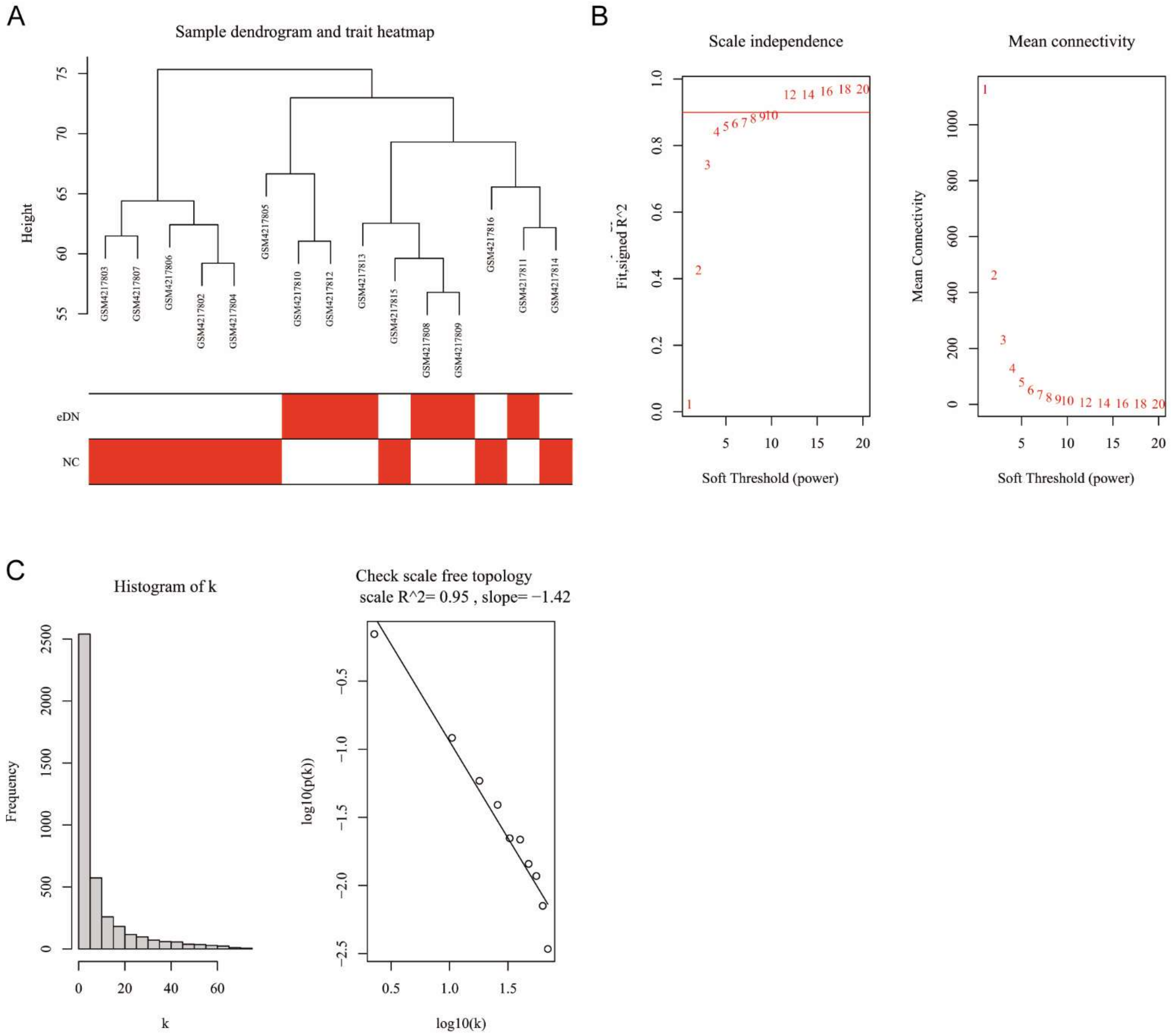

Supplementary Figure1. Determination of the soft threshold power of the WGCNA in the eDN and NC group. (A) Sample clustering dendrograms. (B) Analysis of the scale-free index for a set of soft-thresholding powers (β). (C) Histogram of connectivity distribution when β=12 and checking the scale free topology.

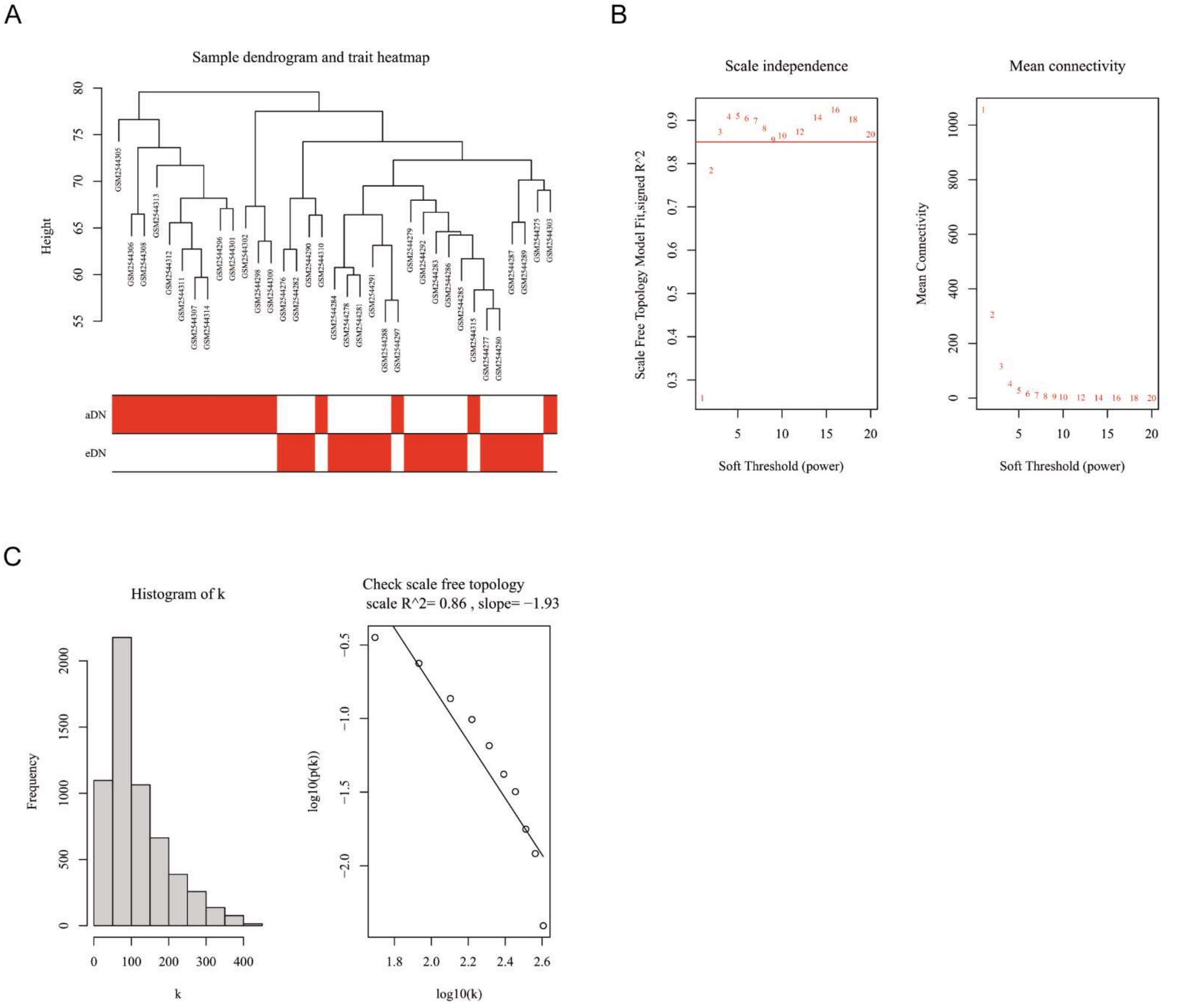

Supplementary Figure 2. Determination of the soft threshold power of WGCNA in the aDN and eDN groups. (A) Sample clustering dendrogram. (B) Scale-free index analysis of a set of soft threshold powers (β). (C) Distribution histogram of connectivity when β = 9 and inspection of the scale-free topological structure.

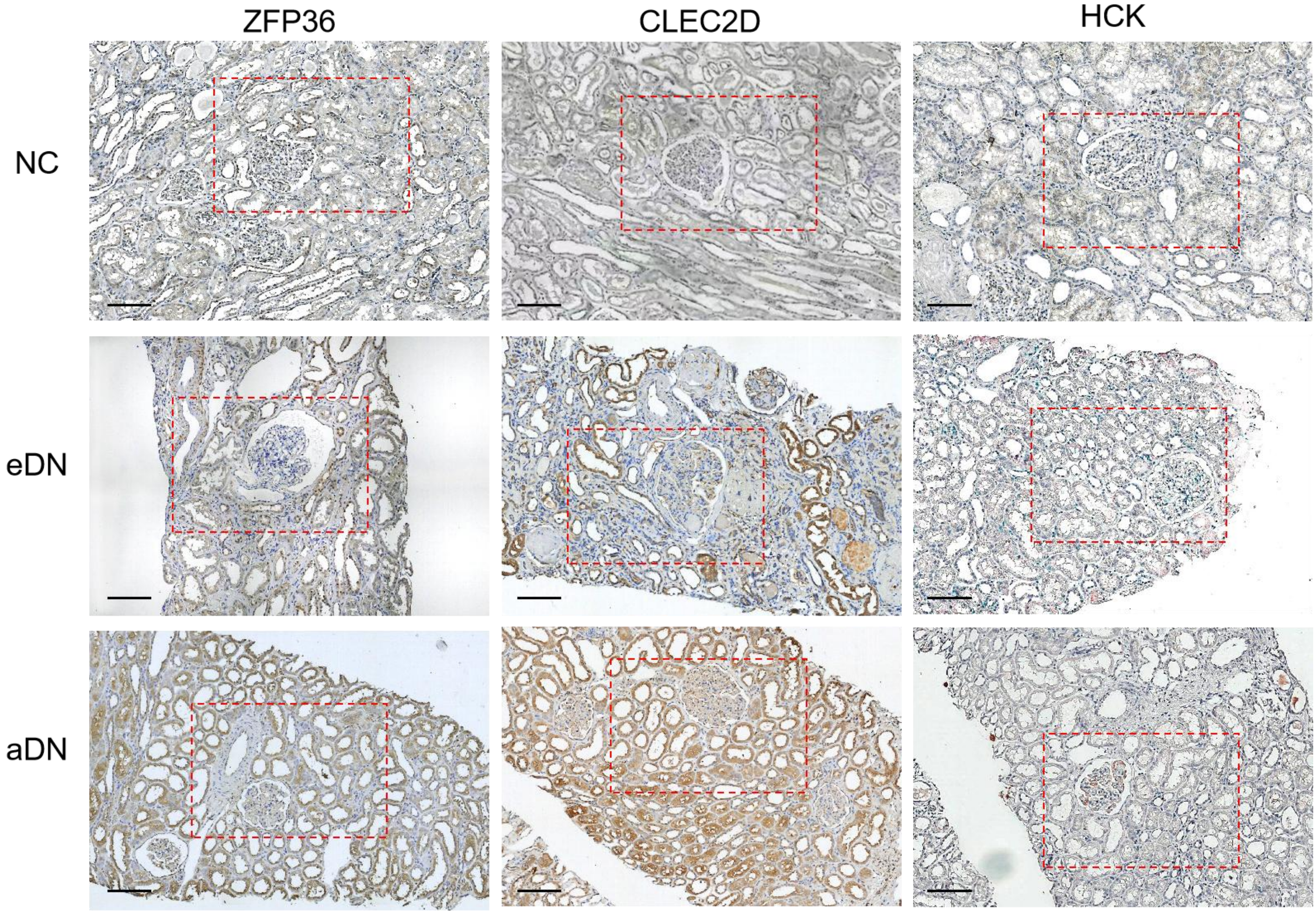

Supplementary Figure 3. Representative immunohistochemical images of ZFP36, CLEC2D, and HCK in renal tissues from DN patients at different magnifications (magnification: ×200, scale bar: 100μm). The corresponding area in the high-magnification (×400) image in the main text is marked with a red box.

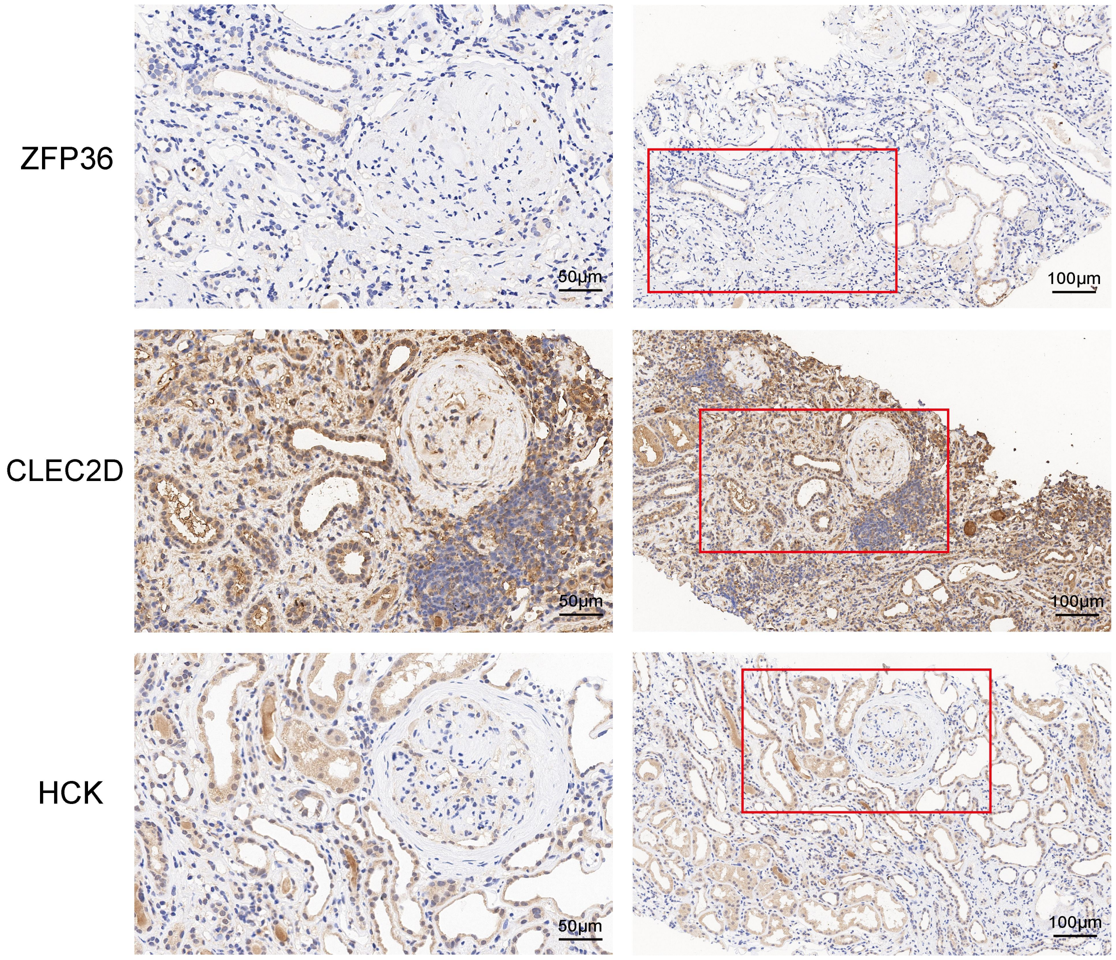

Supplementary Figure 4. Immunohistochemical staining of ZFP36, CLEC2D, and HCK in renal tissues from DN patients across different microscopic fields (magnification: ×200 on the left; ×400 on the right). The specific area corresponding to the high-magnification image on the left was marked with red boxes.

A

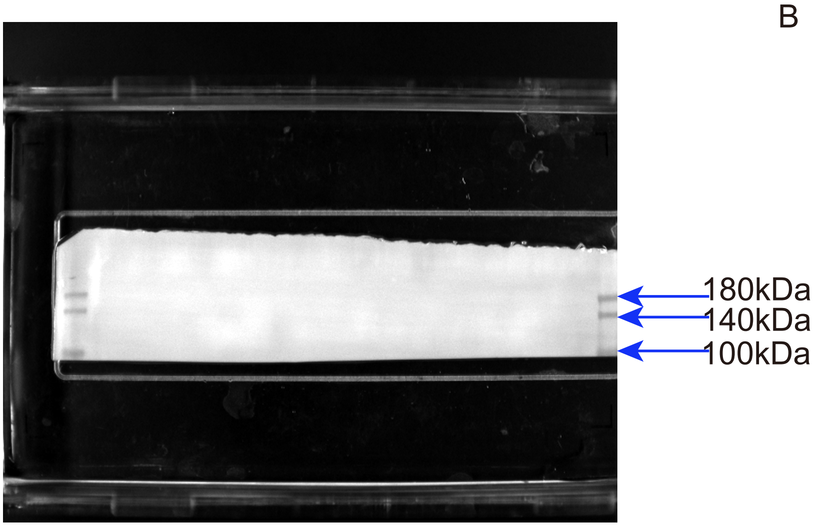

NLRP3 (bright field)

B

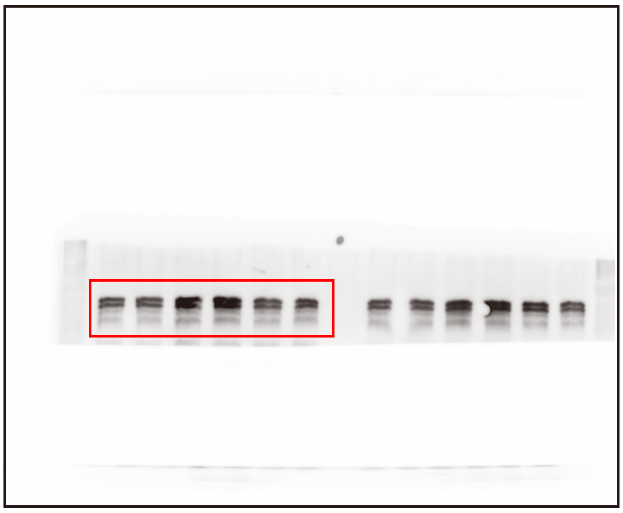

NLRP3 (left lane was shown in the manuscript)

C

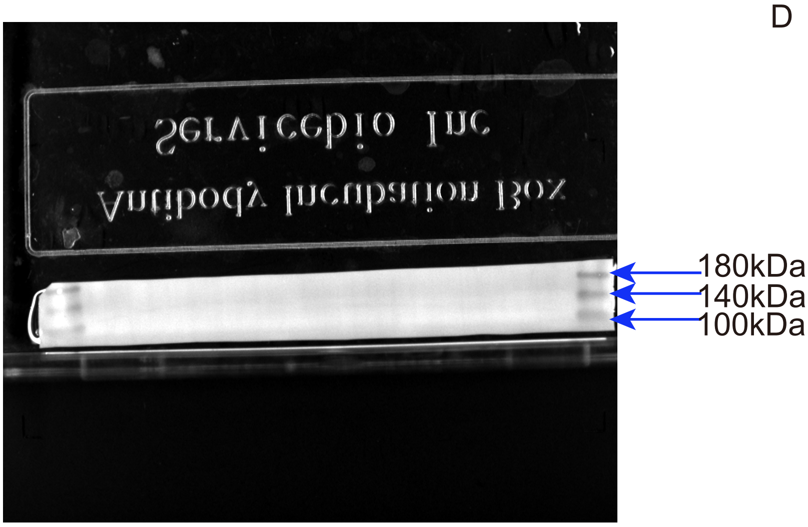

Vinculin (bright field)

D

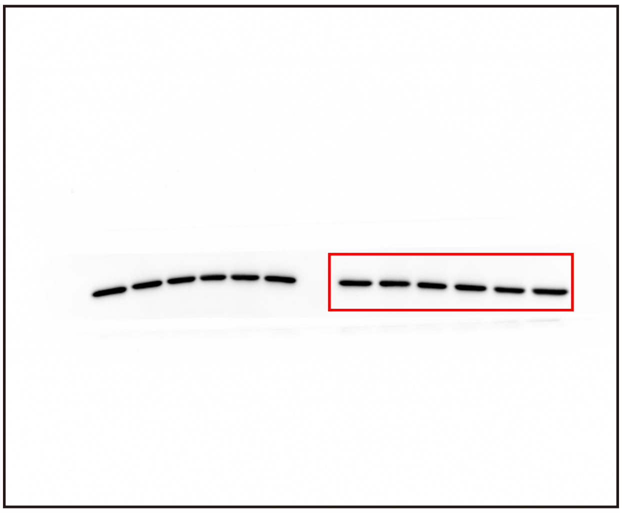

Vinculin (right lane was shown in the manuscript)

E

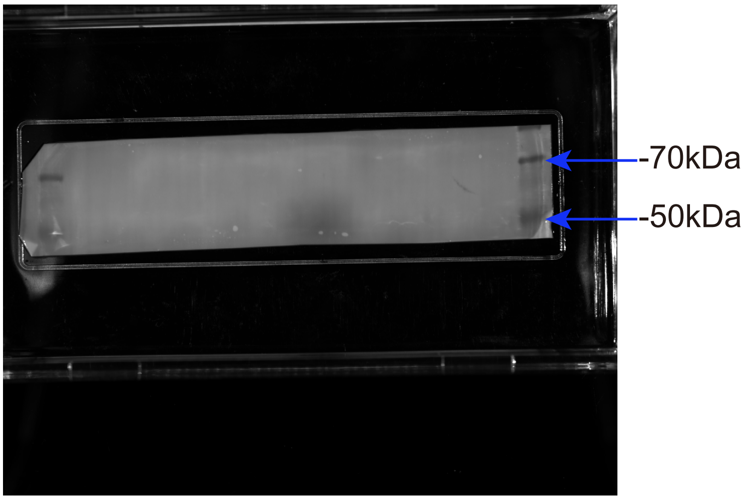

HCK (bright field)

F

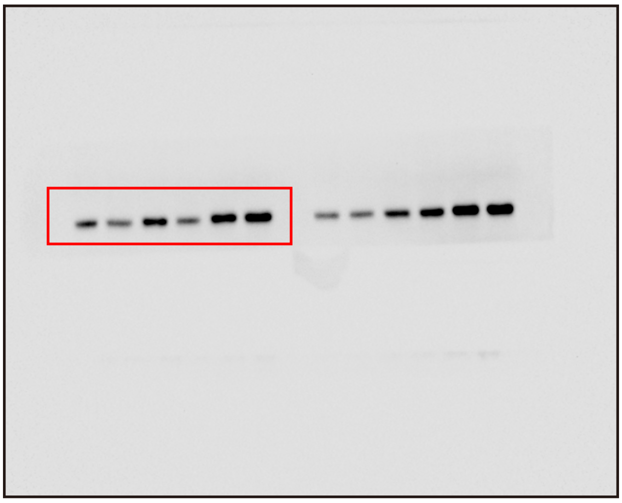

HCK (left lane was shown in the manuscript)

G

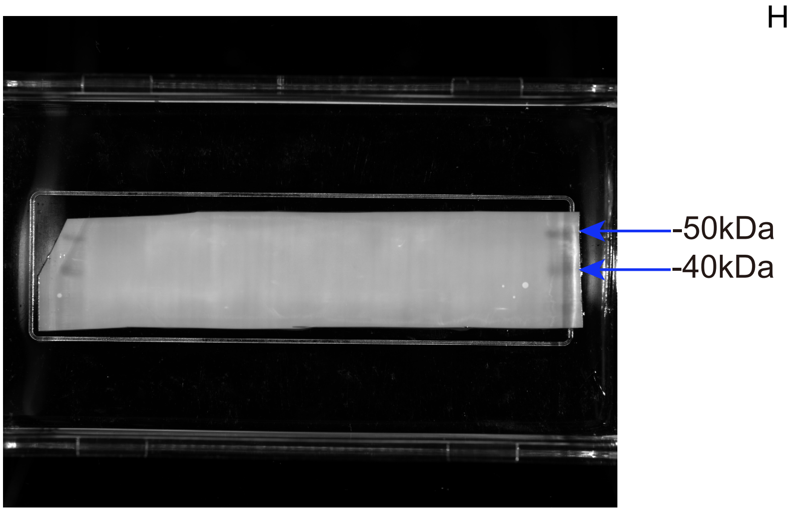

ZFP36 (bright field)

H

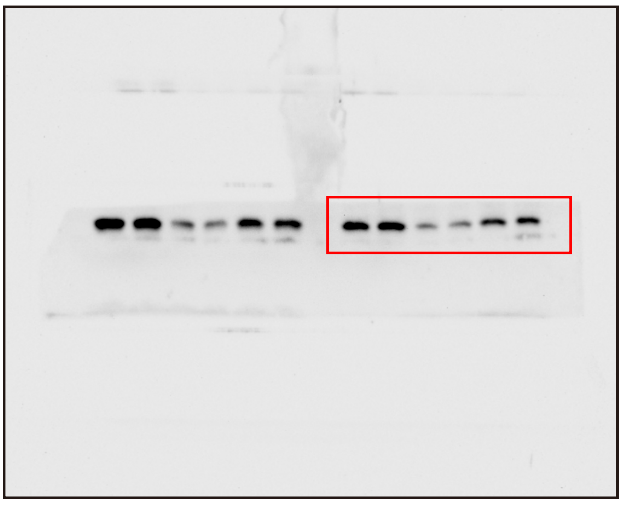

ZFP36 (right lane was shown in the manuscript)

I

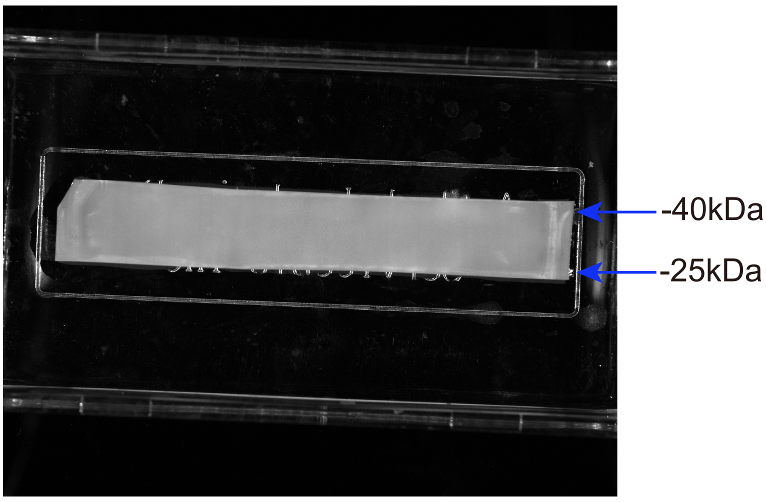

CLEC2D (bright field)

J

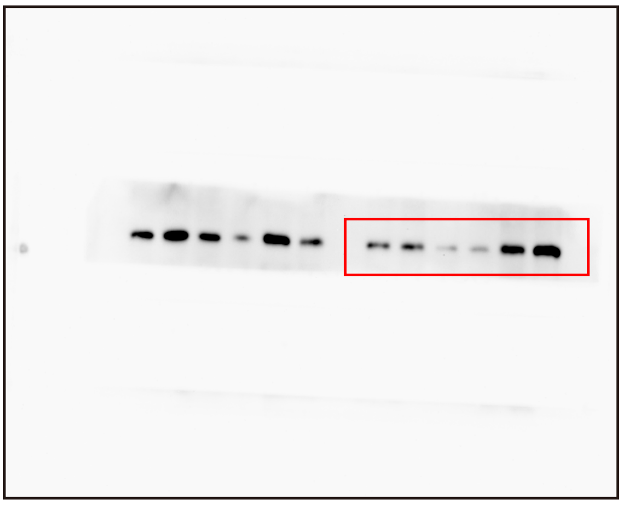

CLEC2D (right lane was shown in the manuscript)

K

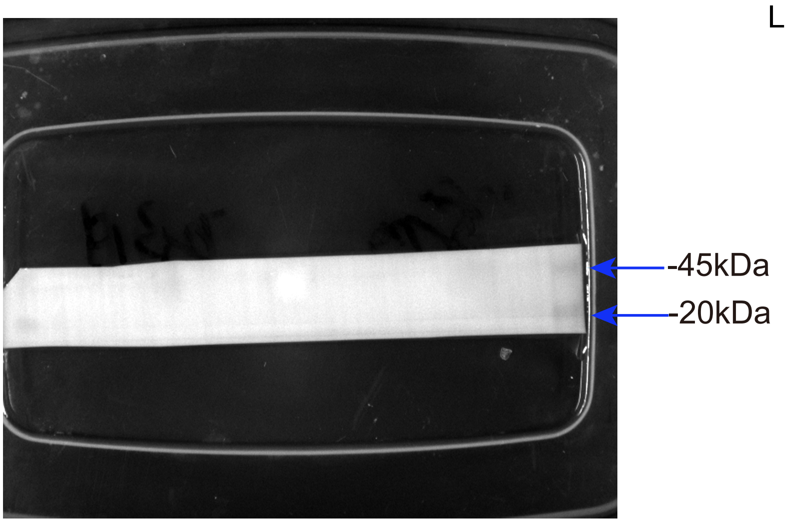

Caspase-1 (bright field)

L

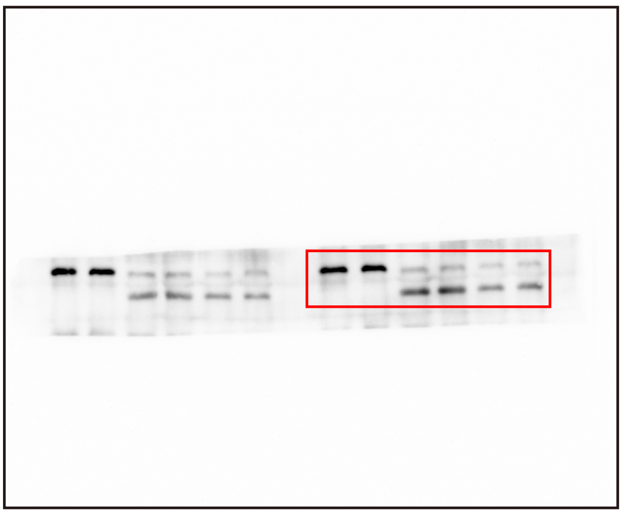

Caspase-1 (right lane was shown in the manuscript)

M

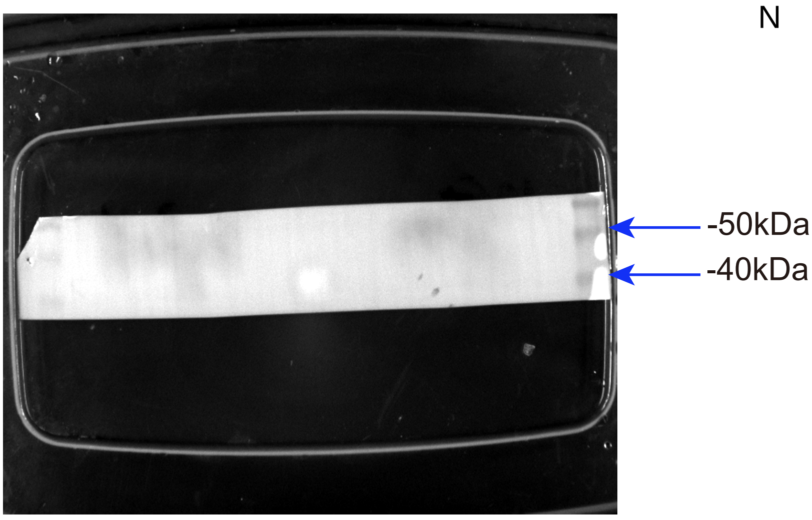

β-actin (bright field)

N

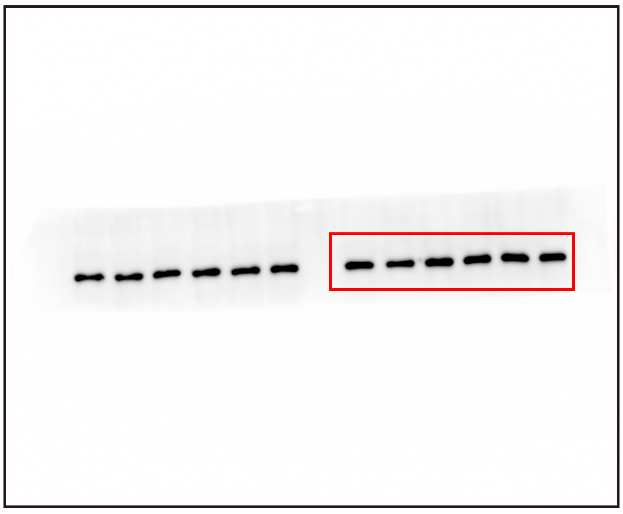

β-actin (right lane was shown in the manuscript)

Supplementary Figure 5. Original Western blot images and annotations. Bright-field photos (A, C, E, G, I, K, M) with prestained marker labels and the corresponding chemiluminescent images (B, D, F, H, J, L, N) are presented.
